# Vitamin D and COVID-19: Inmunomodulatory Effects and the Mexican Public Health Perspectives

**DOI:** 10.3390/ijms27177510

**Published:** 2026-08-22

**Authors:** María Luisa Muñoz-Almaguer, Erick Yair Flores-Salas, Carlos Bancalari-Organista, Raymundo Escutia-Gutierrez, María Virgen-Montelongo, Felipe Alexis Avalos-Salgado, Esmeralda Marisol Franco-Torres, Ana Montserrat Corona-España

**Affiliations:** 1 Department of Pharmacology, University Center of Applied Sciences and Engineering, University of Guadalajara, Guadalajara 44100, Mexico; erick.flores1309@alumnos.udg.mx (E.Y.F.-S.); carlos.bancalari@academicos.udg.mx (C.B.-O.); raymundo.escutia@academicos.udg.mx (R.E.-G.); maria.virgen@academicos.udg.mx (M.V.-M.); alexis.avalos@academicos.udg.mx (F.A.A.-S.); esmeralda.franco@academicos.udg.mx (E.M.F.-T.); 2 Department of Chemistry, University Center of Applied Sciences and Engineering, University of Guadalajara, Guadalajara 44100, Mexico; ana.corona@academicos.udg.mx

**Keywords:** Vitamin D, SARS-CoV-2 virus, cytokines, COVID-19, adjuvant

## Abstract

Vitamin D is a fat-soluble vitamin, considered a prohormone, that plays a fundamental role in calcium absorption and reabsorption, as well as the modulation of inflammatory response in infectious conditions such as COVID-19. A literature search was conducted in scientific databases and official sources, selecting studies published in the last 10 years in Spanish and English related to vitamin D and COVID-19; studies unrelated to the nutritional approach were excluded to reduce bias and ensure a rigorous and reproducible analysis. It was observed that the probability of contracting COVID-19 increases as the D3 hypovitaminosis prevalence increases. In Mexico, the relevance of vitamin D deficiency or supplementation in this disease has been controversial. Some studies claim that vitamin D supplementation could be a protective factor. According to the National Health Survey in Mexico (Ensanut), the Mexican population has significant deficiencies in this vitamin, contributing to an unfavorable diagnosis, particularly for children and pregnant women, as it is considered a deficiency in the blood when it is below 30 ng/mL and insufficiency when below 20 ng/mL, making these deficiencies important risk factors in the development of COVID-19 severe cases. Nevertheless, the use of vitamin D as an adjuvant agent in the treatment of COVID-19 can represent one of the main options for public health, providing therapeutic properties and health benefits.

## 1. Introduction

The World Health Organization (WHO) registered a new coronavirus (SARS-CoV-2) at the end of 2019 in the city of Wuhan in China [1]. COVID-19 is a respiratory disease caused by a new positive-sense single-stranded ribonucleic acid (RNA) beta coronavirus with a 30 kb genome and at least 50 open reading frames, which allow coding of 50 proteins with non-structural, structural and accessory functions that intervene in the biological functions of the virus [2].

Of these structures, the virus’s spiked glycoprotein plays a crucial pathogenic role by specifically binding to the angiotensin-converting enzyme 2 (ACE2) on host cells, using it as its primary receptor for cellular entry. This interaction induces internalization and subsequent downregulation of surface ACE2, disrupting pulmonary tissue homeostasis [3,4].

In this context, a series of preventive measures were established, including the use of masks, social distancing, and quarantines, among others. Nevertheless, there was a growing interest from the scientific community to investigate new alternative methods such as nutritional supplementation of vitamin D given its potential pleiotropic capacity around a possible immunomodulatory effect on viral infections of the respiratory tract [5,6,7,8]. This led to the proposal of two possible pathways of interaction between this vitamin and the risk of COVID-19. The first is related to the modulation of inflammatory systems at the level of pulmonary T cells with anti-inflammatory potential. In particular, it has been observed that vitamin D supplementation can attenuate cytokine storm by increasing anti-inflammatory mediators such as IL-10 and stimulating interferon gamma (IFN-γ). Furthermore, it has a direct impact on both the adaptive and innate cellular immune responses, promoting the restoration of central memory CD4+ T-cell subpopulations and significantly enhancing the antiviral cytotoxic response through NK cell degranulation (CD107a) [5,9,10,11]. The second pathway is related to the regulation of angiotensin through the renin–angiotensin–aldosterone system (RAAS). In this pathway, vitamin D acts as a negative endocrine modulator of the RAAS; by inhibiting renin secretion, it suppresses the harmful ACE/Ang II/AT1R axis and, at the same time, upregulates and stimulates the protective ACE2/Ang-(1-7)/MasR axis. This biochemical rebalancing counteracts the virus-induced toxic accumulation of angiotensin II, thereby mitigating the processes of cellular apoptosis, vasoconstriction, and severe pulmonary inflammation that lead to Acute Lung Injury (ALI) [12,13]. This proposes that a deficient serum level of vitamin D (<20 ng/mL) can increase the symptoms and mortality of infections caused by microorganisms due to the inhibition of the immune system [13].

For these reasons, the dosage of this hormone has become a subject of critical study. While standard moderate doses (2000 IU/day) are aimed at correcting nutritional deficiencies, the use of high pharmacological doses (10,000 IU/day) during the acute phase of hospitalization has demonstrated a strong ability to mitigate the progression of acute respiratory distress syndrome (ARDS). This translates into a direct clinical impact, resulting in dramatic reductions in the length of hospital stay for critically ill patients [3,6,11,14].

Although several systematic reviews have evaluated the relationship between vitamin D and COVID-19, most have synthesized global evidence without considering country-specific factors that may influence vitamin D status, disease severity, and clinical outcomes. Mexico represents a unique setting because of its high prevalence of vitamin D deficiency despite abundant sunlight, together with a high burden of obesity, diabetes, and COVID-19 mortality. To date, no review has specifically examined the available evidence in the Mexican population. Therefore, this review aims to summarize and critically analyze the evidence regarding vitamin D and COVID-19 in Mexico, identify current knowledge gaps, and highlight priorities for future research and public health interventions.

## 2. Biological Aspects of Vitamin D

**Figure 1:**
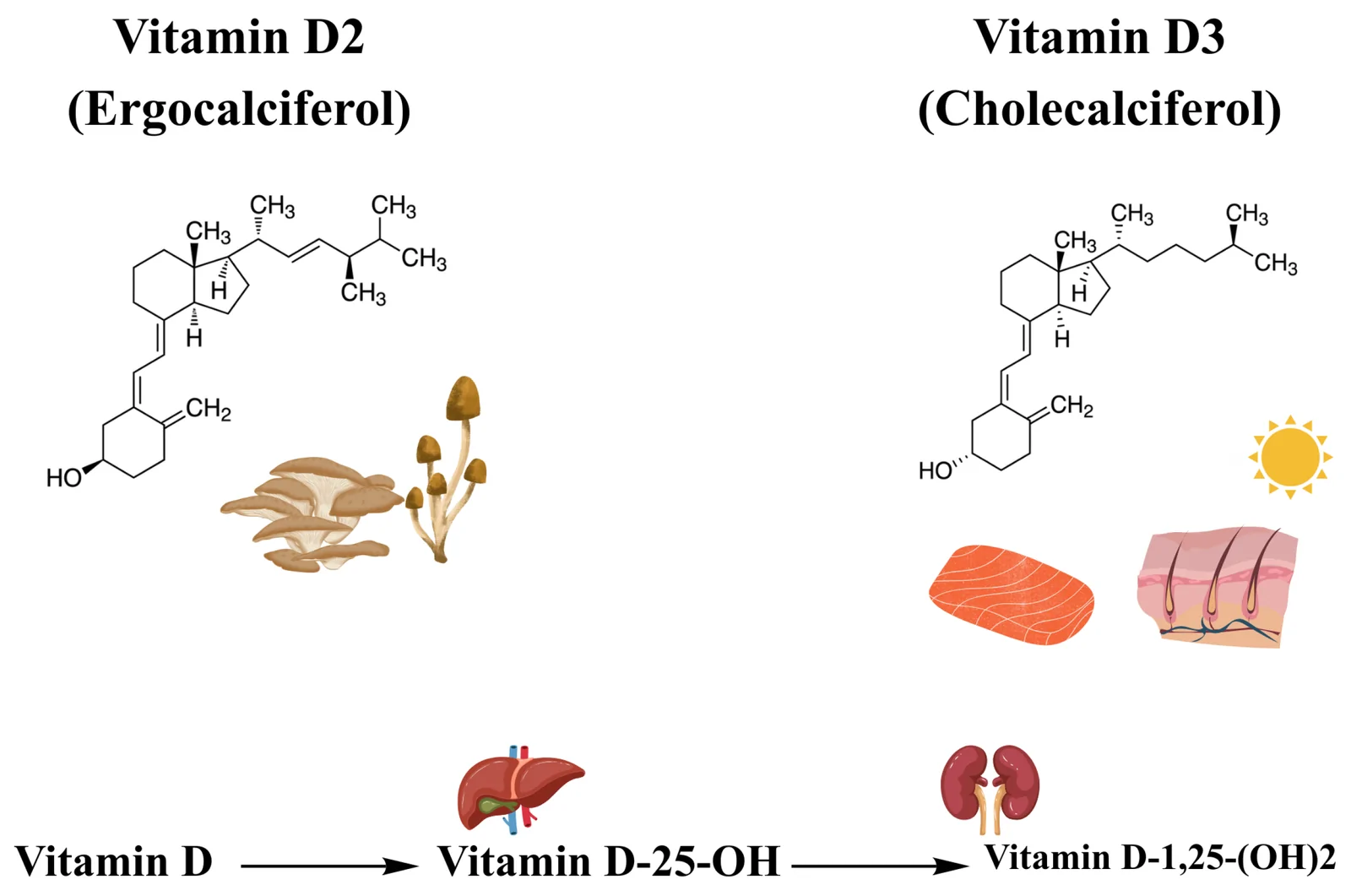
Composition and structure of vitamin D2 and D3, their main source and biotransformation.

Vitamin D belongs to the group of fat-soluble vitamins. It is essential to maintain an adequate concentration of serum calcium and phosphate for bone health and normal muscle function [15]. It is considered a prohormone, whose functions in the absorption and reabsorption of calcium, which contribute to the proper functioning of the immune system, are well known [2]. The main natural source of vitamin D is the synthesis of cholecalciferol in the skin through a photochemical reaction with UV radiation from sun exposure [15]. There are five forms of vitamin D (D1–D5), where vitamin D2 and D3 have been the most studied; vitamin D2 is synthesized endogenously through the diet in the small intestine, while vitamin D3 can also be obtained through exposure to ultraviolet light between 280 and 320 nm (Figure 1) [4,16,17]. During exposure to ultraviolet light, photons are absorbed by 7-dehydrocholesterol, forming precholecalciferol, which is rapidly converted to cholecalciferol. It is then converted into the active form and transported by vitamin D-binding protein (DBP) for activation in the liver and subsequently in the kidney. 1,25-hydroxyvitamin D3 is the main circulating form and the best indicator of vitamin D levels; it is responsible for most biological effects [7,14,18,19].

Vitamin D deficiency is a global concern that is prevalent among the elderly, but persists as a common problem in both children and adults worldwide, arising mainly from insufficient dietary vitamin D intake and inadequate sunlight exposure, which is associated with musculoskeletal disorders [15,20,21,22].

### 2.1. Immunomodulatory Mechanisms

**Figure 2:**
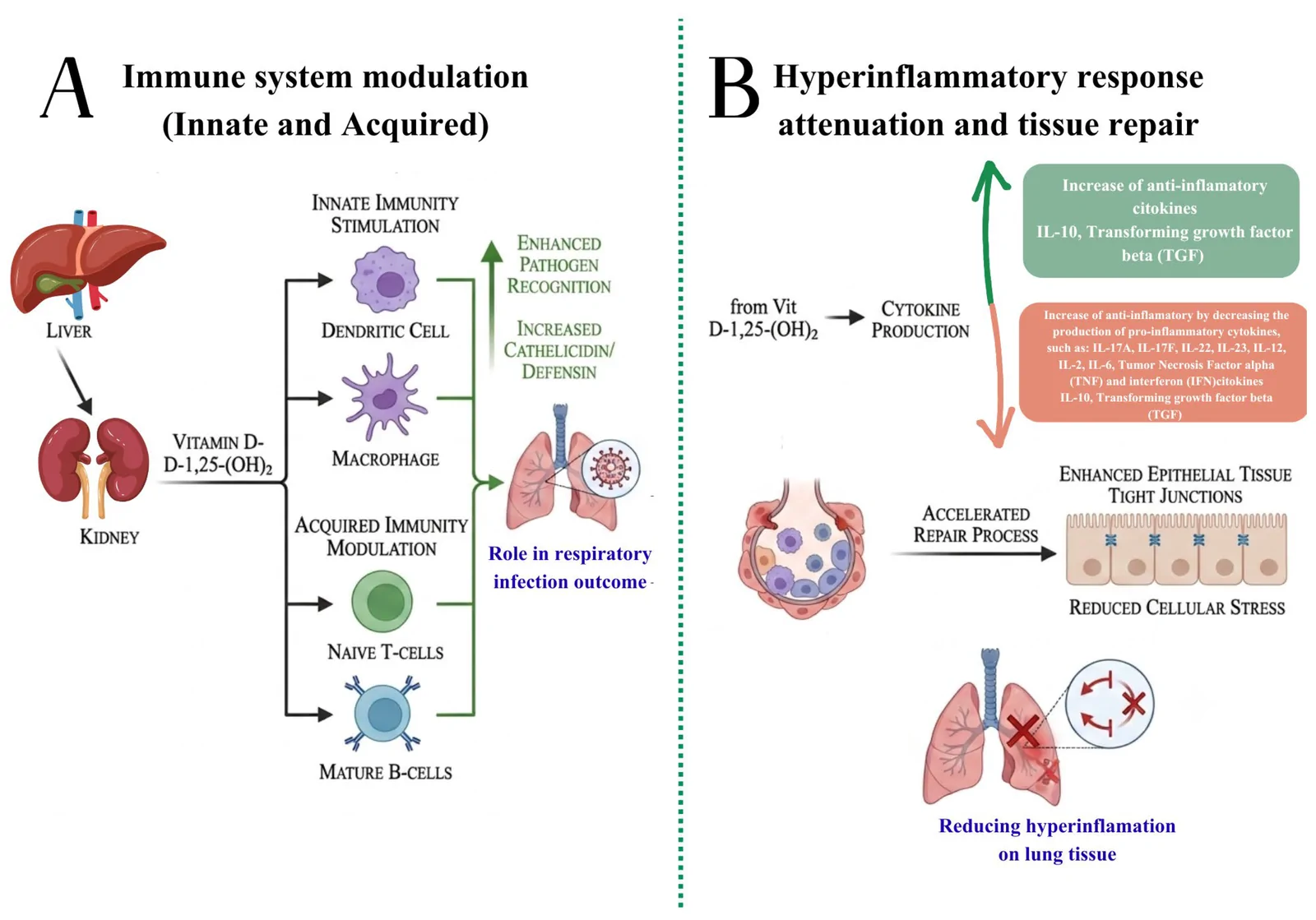
Role of active vitamin D in respiratory infection outcomes, where (**A**) corresponds to the innate and acquired immune system modulation via antigen-presenting cells and lymphocytes and (**B**) attenuation of the hyperinflammatory cascade through cytokine balance shift and alveolar epithelial tissue repair [7,9,10,11,24,25,26].

Vitamin D has emerged as an important regulator of immune homeostasis, extending far beyond its classical role in calcium and bone metabolism. Its immunological effects are primarily mediated through activation of the vitamin D receptor (VDR), a nuclear transcription factor expressed in most innate and adaptive immune cells, including macrophages, dendritic cells, T lymphocytes, B lymphocytes, and natural killer (NK) cells [9,10,12,14,23,24]. Upon binding to its active metabolite, 1,25-dihydroxyvitamin D3 (calcitriol), the VDR regulates the transcription of numerous genes involved in antimicrobial defense, inflammatory signaling, and immune tolerance. Within the innate immune system, vitamin D enhances the antimicrobial capacity of macrophages and monocytes by promoting phagocytosis and inducing the expression of antimicrobial peptides such as cathelicidin (LL-37) and β-defensins. It also modulates dendritic cell maturation by reducing the expression of MHC class II molecules and co-stimulatory proteins (CD40, CD80, and CD86), thereby limiting excessive antigen presentation and inflammatory activation [12,14,24]. In adaptive immunity, vitamin D shifts the immune response toward a more tolerogenic phenotype. It suppresses Th1 and Th17 differentiation while promoting regulatory T cells (Tregs), resulting in reduced activation of autoreactive immune responses [7,9,23]. Furthermore, vitamin D inhibits B-cell proliferation, plasma cell differentiation, and immunoglobulin production, contributing to the control of excessive immune activation [12,14,24]. These immunomodulatory actions are particularly relevant in SARS-CoV-2 infection. Severe COVID-19 is characterized by dysregulated immune activation, exaggerated production of pro-inflammatory cytokines, endothelial dysfunction, and diffuse lung injury [7,9,25]. By limiting excessive activation of Th1/Th17 responses while promoting regulatory pathways, vitamin D has been proposed to reduce the risk of cytokine storm and subsequent tissue damage. Experimental studies have shown that vitamin D downregulates the production of several pro-inflammatory cytokines, including IL-6, IL-2, IL-12, IL-17, TNF-α, and IFN-γ, while increasing anti-inflammatory mediators such as IL-10 and TGF-β, as shown in Figure 2 [7,9,10,11,24,25,26]. This cytokine profile favors the resolution of inflammation and may facilitate epithelial repair, particularly in pulmonary tissue [7,9,25].

At the molecular level, the immunomodulatory effects of vitamin D depend on activation of the vitamin D receptor (VDR), which forms a heterodimer with the retinoid X receptor (RXR). This VDR-RXR complex binds to vitamin D response elements (VDREs) within target genes, regulating the transcription of numerous genes involved in immune function, inflammation, antimicrobial defense, and epithelial integrity [3,12,27]. In addition, several immune cells, including macrophages, dendritic cells, and activated T and B lymphocytes, express the enzymes 25-hydroxylase and 1α-hydroxylase, enabling the local conversion of circulating vitamin D metabolites into the active hormone calcitriol. This intracrine signaling allows immune cells to locally regulate inflammatory responses independently of renal vitamin D activation. Furthermore, vitamin D promotes macrophage polarization toward the anti-inflammatory M2 phenotype while suppressing the pro-inflammatory M1 phenotype, contributing to the resolution of inflammation and tissue repair [7,9,23].

Although these biological mechanisms provide a plausible rationale for the potential protective role of vitamin D during COVID-19, most evidence derives from experimental models, in vitro studies, and observational research. Whether these immunological effects translate into clinically meaningful benefits remains uncertain because randomized controlled trials have produced inconsistent results. Therefore, the mechanistic evidence should be interpreted as biologically plausible rather than definitive proof of therapeutic efficacy.

### 2.2. Vitamin D and Viral Inflammation

Viruses are capable of producing inflammation, where the first thing that stands out is the excessive presence of leukocytes and the activation of resident leukocytes in the tissues. Generally, when we talk about inflammation associated with viral processes, we can find two types: systemic inflammation, which reflects an excessive activation of circulating leukocytes in response to viremia, and clinical inflammation, which can alleviate or aggravate the disease, depending on the patient. Currently, there are several relevant virulent viruses such as rabies, smallpox, Ebola, Marburg and Nipah viruses, hantavirus, some influenza viruses, human immunodeficiency virus (HIV), severe acute respiratory syndrome coronavirus (SARS-CoV), Middle East respiratory syndrome (MERS)-CoV and SARS-CoV-2, the most recent addition to the list [20,22,28,29].

To understand this phenomenon, it is important to know about a process that occurs in harmony with the inflammation cascade, the hemostatic system. This system, through endothelial dysfunction, platelet and coagulation activation, promotes thrombosis, which is called thromboinflammation, and the inflammasome, which promotes innate and adaptive immune responses. The latter plays an important pathophysiological role in pathologies that present with inflammatory and thrombotic phenomena, such as COVID-19, where a cytokine storm has been described as one of the responsible mechanisms and where the survival of infected organisms depends on their ability to promote a rapid and effective response against infection, hemorrhage and tissue injury, thanks to the concurrence of innate defense mechanisms, such as the hemostatic system and the immune system [3,26,30].

As mentioned before, vitamin D may decrease the risk of infections by several mechanisms, including maintaining the integrity of a physical barrier by stimulating genes encoding proteins related to cell integrity and junctions such as occludin (tight junctions), connexin 43 (gap junctions), and E-cadherin (adherent junctions), or through enhancing innate and/or adaptive cellular immunity. Specialized defense cells such as macrophages, monocytes, dendritic cells, and T and B lymphocytes express VDR and enzymes for the synthesis of vitamin D through the induction of antimicrobial peptides such as cathelicidin and beta-2-defensin. In addition, it contributes to reducing the so-called cytokine storm that occurs in severe viral infections, such as COVID-19, after inhibiting the production of proinflammatory cytokines from Th1 cells such as tumor necrosis factor alpha (TNF-) and interferon gamma (INF-) [7,9,10,11,31].

Recent studies have shown that it is common to find low levels of vitamin D in elderly people as a result of aging-associated changes in vitamin D and calcium and metabolism, that is, the capacity to absorb calcium from circulating 1,25(OH)2D is reduced, and therefore, the renal production of this by the aged kidney is reduced, which leads to a low production of vitamin D by the skin [14,21,32,33].

### 2.3. Epidemiological Evidence in Mexico

Since the end of 2023, it has become a priority for academics and researchers to study the impact of the COVID-19 pandemic in Mexico, learn lessons, and avoid future catastrophes [34]. Currently, COVID-19 can be considered an endemic disease, which means that it is constantly present in the population and particular geographic area, without reaching epidemic levels. This implies that the disease is not transitory or sporadic, but remains stable in that area. Therefore, epidemiological surveillance of diseases such as COVID-19, influenza, and other respiratory viruses focuses mainly on the immediate detection of cases that meet the operational definition of suspected cases in order to contain the spread of the virus in the Mexican population and trigger epidemiological and laboratory surveillance, care, prevention, and control actions [20,22,35].

Table 1 presents the temporal evolution and demographic and clinical stratification of confirmed SARS-CoV-2 (COVID-19) cases in Mexico between 2020 and 2024. The table summarizes sex distribution, clinical severity, hospitalization patterns, intensive care unit (ICU) requirement, mortality, and epidemiological context across different phases of the pandemic, including the emergence of variants and the vaccination rollout period.

**Figure 3:**
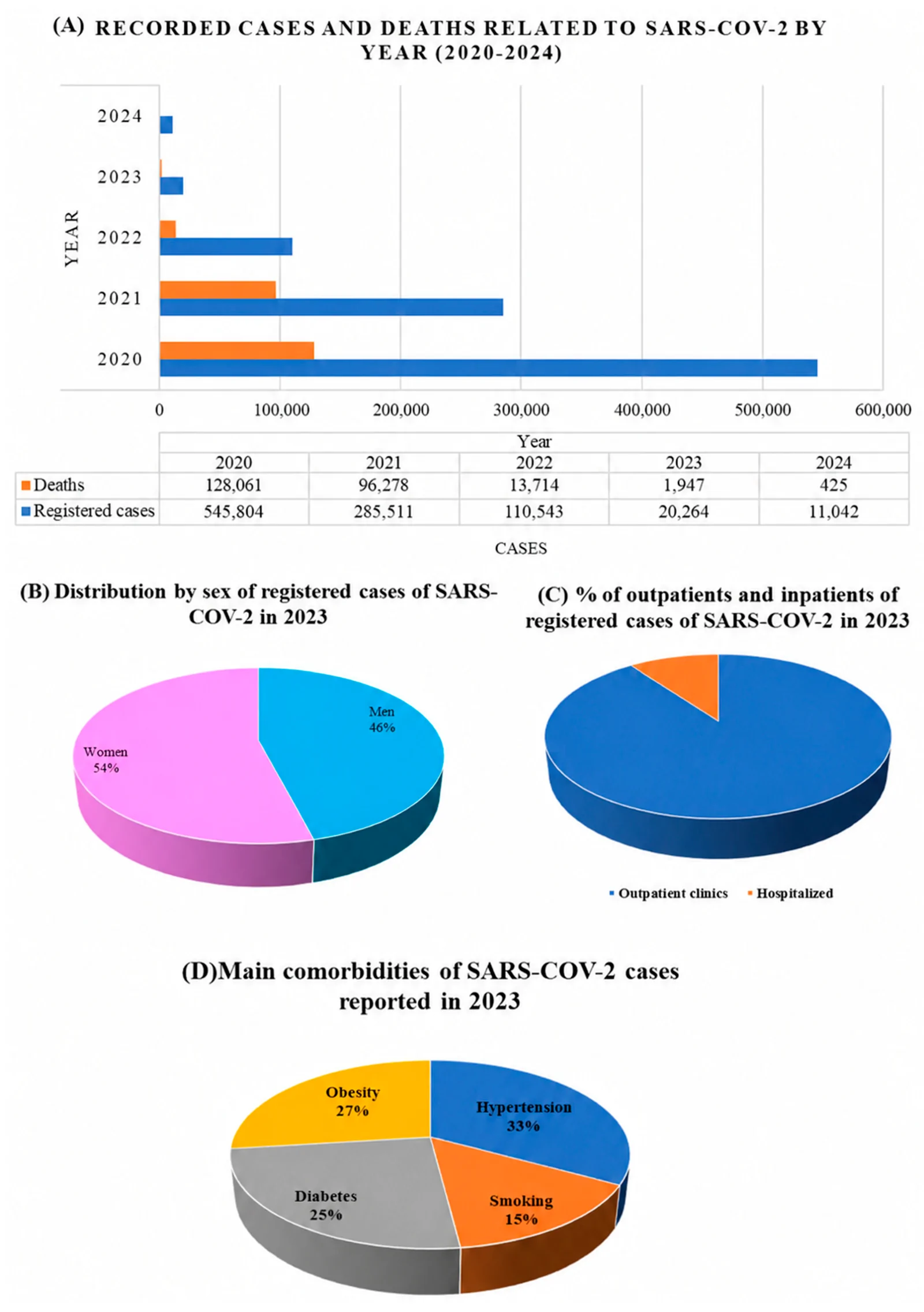
Statistics in Mexico, where (**A**) shows the reported cases and deaths recorded by SARS-CoV-2 from 2020 –2024, while (**B**) presents the distribution by sex of the cases recorded by SARS-CoV-2 in 2023, (**C**) represents the distribution of hospitalized and outpatient patients by SARS-CoV-2 in 2023 and (**D**) the main comorbidities recorded for SARS-CoV-2 in 2023 [35,36].

**Figure 4:**
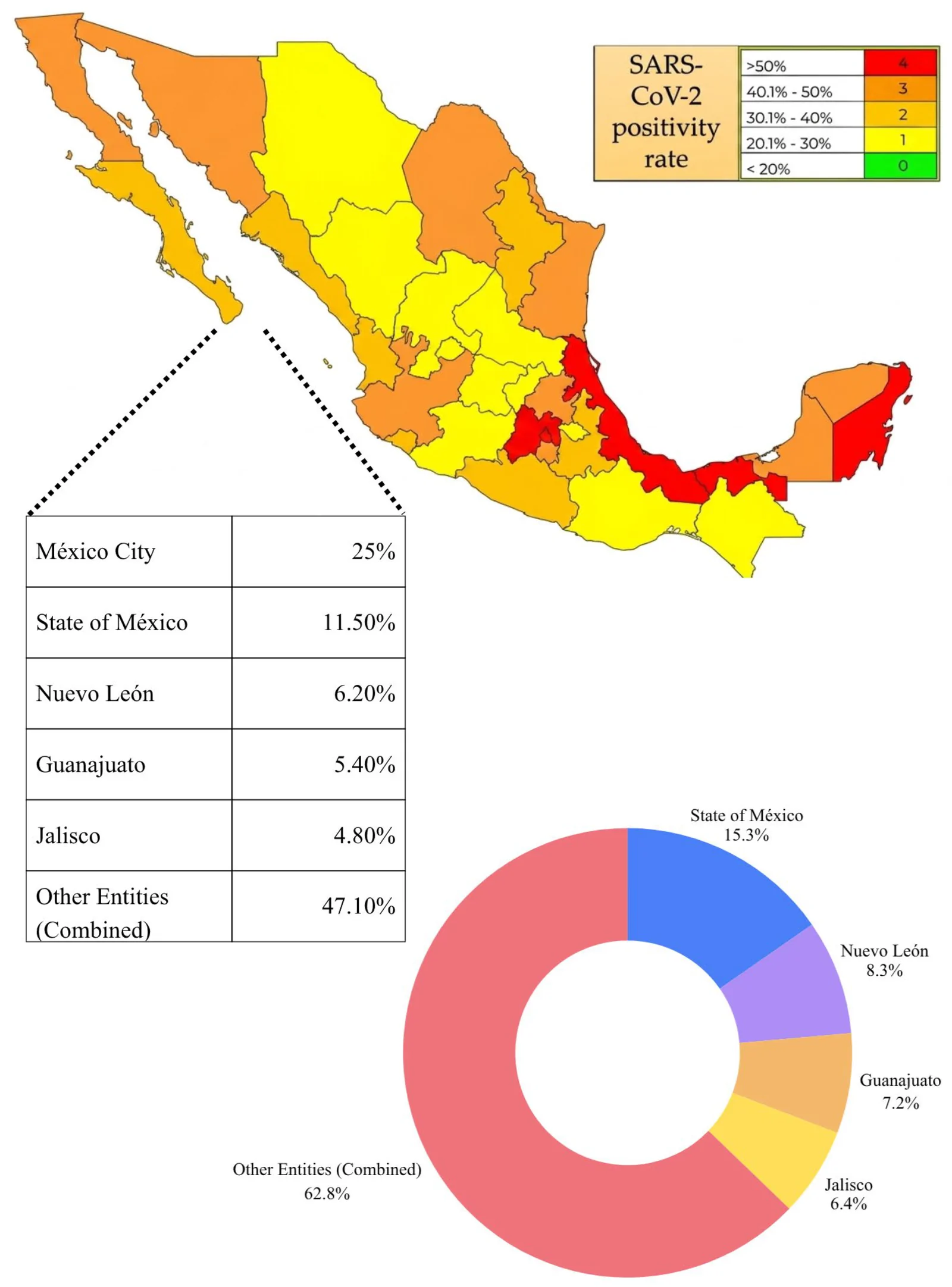
Map of SARS-CoV-2 positivity percentage by federal entity, where the red areas represent the states with the highest incidence of cases >50%, orange an incidence of 40.1–50%, dark yellow of 30.1–40%, light yellow of 20.1–30%, while the green color represents an incidence < 20% according to the COVID-19/Mexico Report of 30 March 2024 [35,36].

Herein, we aimed to understand the clinical–epidemiological pattern of SARS-CoV-2 in Mexico within four years following the onset of the pandemic. As shown in Table 1 and Figure 3, the highest number of reported cases and deaths was observed during 2020 and 2021, which progressively declined through 2024. In Figure 4, the choropleth map illustrates regional differences in SARS-CoV-2 positivity rates by federal entity, based on the COVID-19/Mexico Report from March 30, 2024. Positivity rates were classified into five categories: <20% (green), 20.1–30% (light yellow), 30.1–40% (dark yellow), 40.1–50% (orange), and >50% (red). States shown in red represent the highest positivity rates, indicating the greatest burden of infection, whereas green areas correspond to the lowest positivity rates. Higher positivity was primarily concentrated in several coastal and southeastern states, particularly Veracruz and Quintana Roo, highlighting persistent regional disparities in SARS-CoV-2 transmission during the study period [36].

In Mexico, the relevance of vitamin D deficiency or supplementation in this disease has been controversial. Some studies claim that vitamin D supplementation could be a protective factor and it is considered a deficiency in the blood when it is below 30 ng/mL and insufficiency when below 20 ng/mL [9]. Its deficiency seems to be an important risk factor in the development of severe cases of COVID-19. Studies show that in populations where the prevalence of vitamin D3 hypovitaminosis is high, there is a greater probability of contracting COVID-19 [14,15,21,37]. Vitamin D supplementation is considered an adjuvant therapy for patients with COVID-19. Having high circulating levels of 25OH-D delays the progression of the disease and improves patient survival, indicating the good tolerance and safety of high doses of Vitamin D [6,11,38].

The Mexican Social Security Institute (IMSS) highlighted that there is still no study with conclusive results regarding whether vitamin D supplementation modulates the response to the SARS-CoV-2 virus and does not cause the severe form of the disease, which is why the IMSS Medical Nutrition Research Unit is carrying out controlled clinical trials [39,40,41].

Different clinical studies, where hundreds of patients infected with SARS-CoV-2 were included, were carried out, with different doses and regimens of Vitamin D, to evaluate the evolution of each of them. Rodríguez et al., in 2020 [42], carried out a retrospective, observational, case–control, analytical and cross-sectional study at the Central Military Hospital of Mexico that included hospitalized patients whose serum concentration of vitamin D was determined before hospital admission to find any direct relationship between serum levels of vitamin D before hospital admission and the mortality rate of patients [33,41,42]. Another study led by Martínez et al. in 2021 showed that in a study of patients hospitalized for COVID-19, vitamin D insufficiency < 30 ng/mL was present in 75% of the population and in 85% of all patients requiring intensive care [25]. This high prevalence of hypovitaminosis D in Mexican critical care settings strongly correlates with global meta-analytical data such as Meng et al., 2023, which demonstrates that vitamin D supplementation significantly reduces the risk of ICU admission (RR 0.63) and the need for mechanical ventilation (RR 0.58), showing a pivotal clinical impact precisely in patient populations with marked baseline deficiencies (RR 0.76) [6,20,21].

Mansur et al., in their 2020 study, proposed that supplementation with 1,25-dihydroxyvitamin D suppresses the recruitment of eosinophils and lymphocytes in the airways, decreasing IL-4 production by T cells, and inhibits T cell migration, attenuating the inflammatory response, working as an adjuvant for other therapies, such as allergen immunotherapy. Also, they proposed that a simultaneous administration of vitamin D and dexamethasone in steroid-resistant asthmatic patients increased IL-10 synthesis to levels similar to those found in steroid-sensitive patients treated with dexamethasone alone [31]. This immunological synergy materializes in acute respiratory distress syndrome (ARDS) secondary to COVID-19; contemporary clinical trials have shown that high-dose vitamin D regimens (10,000 IU/day) significantly upregulate the anti-inflammatory cytokine IL-10 and downregulate cellular damage, dramatically shortening hospital stays from 29.2 to 8.0 days in severe patients, thereby validating the role of vitamin D as a critical immunomodulatory adjuvant alongside standard corticosteroid therapy [5,11,43].

A study carried out in 2011 using data from the 2006 National Health and Nutrition Survey (ENSANUT) assessed vitamin D levels in Mexico. Among the most relevant findings, it was found that 61% of children aged 2 to 12 years had sufficient levels of vitamin D, while 16% had deficiency and 23% insufficiency. Deficiency was more common in preschool children in urban areas. In adolescents aged 13 to 19 years, 69% had sufficient levels, while 8.1% had deficiency and 23% insufficiency. In adults, 70% had sufficient levels, with a greater deficiency observed in rural areas, with 9.8%, compared to urban areas, with 20% insufficiency [4,17,19,36].

Following the same line, another study was carried out by Cruz et al. in 2022 [41], where they analyzed the results of the 2022 National Health Survey (ENSANUT), obtaining that vitamin D deficiency was present in 4.7% of preschoolers and 23.3% of schoolers, as well as in 37.7% of non-pregnant women. The lowest vitamin D deficiency was observed in preschoolers compared to other age groups, while the highest prevalence was observed in boys and girls from urban areas compared to rural ones. In schoolchildren, the highest prevalence of vitamin D deficiency was observed in girls, at 32%, and in urban areas, at 27.2%. For non-pregnant women, vitamin D deficiency was higher in those from urban areas at 40.9% than in those from rural areas at 25.7% [41]. The contrast between ENSANUT 2006 and 2022 highlights a progressive nutritional transition in Mexico, where vitamin D deficiency has risen substantially, particularly within urban centers [4,17]. In the current endemic landscape of COVID-19 in Mexico [14,20,21,35], this widespread hypovitaminosis represents a major public health vulnerability. Given that current epidemiological and clinical evidence confirms that low vitamin D levels significantly increase the risk of severe disease progression [5,6,19,37], this urban deficit underscores the need for targeted nutritional interventions and timely supplementation strategies within Mexican public health frameworks.

The COVID-19 pandemic had a significant impact on global public health, mainly in countries like Mexico, where external factors have influenced the evolution of the disease [1,34,35,36]. Among them, one of the factors that has generated the greatest interest is vitamin D deficiency, since this micronutrient is essential for the regulation of the immune system, and its deficiency has been associated with a higher risk of severe respiratory infections and complications, where COVID-19 stands out [2,14,16,20,21,22,37]. As was mentioned before, vitamin D helps in the modulation of the immune response through the production of cytokines and the activation of T cells and macrophages [2,9,10,18,23,28]. Several studies have suggested that patients with low levels of vitamin D experience a greater susceptibility to developing severe forms of the disease, due to a deregulated inflammatory response and a lower ability to confront viral infection. For this reason, the deficiency of this vitamin has been linked to a higher rate of hospitalization, intensive care requirements and higher mortality [42].

The National Health and Nutrition Survey (ENSANUT) mentions that a large proportion of the Mexican population has vitamin D deficiency or insufficiency, with preschool children, school-aged children, and non-pregnant women being the most vulnerable groups [41]. This is consistent with data reported by the Government of Mexico, indicating that women accounted for the highest proportion of reported COVID-19 cases. These findings suggest a concerning scenario in which factors such as limited sun exposure in certain regions, low dietary intake of vitamin D-rich foods, and sedentary lifestyles may contribute to vitamin D insufficiency and potentially influence health outcomes in the Mexican population [35,36,41,43,44].

Despite this epidemiological context, the available evidence supporting the role of vitamin D in COVID-19 remains heterogeneous. Most published studies are observational and have consistently reported an association between low vitamin D status and poorer COVID-19 outcomes. However, these findings do not establish causality. Likewise, randomized controlled trials have produced inconsistent results, likely due to variations in study design, baseline vitamin D status, supplementation regimens, timing of intervention, and disease severity. Importantly, no randomized clinical trials evaluating vitamin D supplementation for COVID-19 have been conducted in the Mexican population. This lack of country-specific clinical evidence represents an important knowledge gap and underscores the need for well-designed randomized controlled trials that consider Mexico’s epidemiological, nutritional, and demographic characteristics.

## 3. Materials and Methods

### 3.1. Nature of the Review

This work was designed as a narrative review aimed at integrating current evidence regarding the immunomodulatory effects of vitamin D in COVID-19 and its implications for public health in Mexico. Although this review was not conducted as a systematic review, the literature identification and selection process was performed using transparent and reproducible methodological criteria inspired by the PRISMA recommendations whenever applicable.

### 3.2. Information Sources and Search Strategy

This narrative review was conducted to integrate current evidence on the immunomodulatory effects of vitamin D during the COVID-19 pandemic, with particular emphasis on its clinical relevance and implications for public health in Mexico. A literature search was conducted using the electronic databases MEDLINE, PubMed, SciELO, and Elsevier. Additional information was obtained from peer-reviewed academic publications and official reports issued by recognized public health organizations, including the World Health Organization (WHO), the Centers for Disease Control and Prevention (CDC), the Mexican Ministry of Health, and publicly available epidemiological statistics from the Government of Mexico.

The search focused on publications from the previous 10 years (2015–2025) available in English and Spanish. Search terms included combinations of “Vitamin D”, “COVID-19”, “SARS-CoV-2”, “immunomodulation”, “vitamin D deficiency”, “public health”, and “Mexico”, combined with Boolean operators (AND/OR) when appropriate. Priority was given to peer-reviewed publications, including narrative and systematic reviews, meta-analyses, randomized clinical trials, observational studies, case–control studies, and relevant epidemiological reports addressing the relationship between vitamin D and COVID-19.

### 3.3. Study Selection and Thematic Synthesis

Publications were screened according to their relevance to the objectives of this review. Studies primarily addressing the immunological mechanisms of vitamin D, clinical outcomes in COVID-19, therapeutic interventions, or epidemiological evidence relevant to the Mexican population were prioritized. Publications unrelated to vitamin D or that focus on a nutritional rather than an immunological framework were excluded from the final synthesis.

- To facilitate an integrative interpretation of the evidence, the selected literature was analyzed and organized using a thematic and methodological matrix. Rather than quantitatively comparing homogeneous studies, the evidence was grouped according to research design, principal variables investigated, and overall contribution to the understanding of vitamin D in COVID-19. The selected studies were synthesized into four main thematic axes based on their specific research design, primary variables, and level of clinical evidence: Mechanistic and Pathophysiological Immunomodulation: This category clusters basic science research, narrative reviews, and expert perspectives exploring the biochemical and cellular roles of Vitamin D. It focuses on its interaction with the renin–angiotensin system (RAS/ACE2 axis), the regulation of pro-inflammatory cytokines, the inhibition of the inflammasome, and the induction of antimicrobial peptides (e.g., LL37) in SARS-CoV-2 infection.
- Clinical Evidence and Prognostic Outcomes: This axis encompasses observational clinical studies (both prospective and retrospective cohorts, as well as case–control designs) and comprehensive systematic reviews or meta-analyses. These papers evaluate the statistical association between serum 25-hydroxyvitamin D [25(OH)D] levels and clinical outcomes, including infection susceptibility, hospitalization risk, intensive care unit (ICU) admission, and mortality rates.
- Therapeutic Interventions and Clinical Trials: This group comprises randomized controlled trials (RCTs), study protocols, and intervention-specific meta-analyses. It assesses the clinical efficacy, safety profiles, and optimal dosing strategies of Vitamin D supplementation (standard vs. high-dose regimens) as an adjunctive therapeutic or prophylactic agent.
- Public Health, Nutritional Epidemiology, and Socio-Demographical Factors: This section consolidates national epidemiological reports, probabilistic health surveys, institutional guidelines, and population-based regression models. It focuses heavily on the Mexican healthcare framework, assessing the impact of lockdowns on vitamin synthesis, historical nutritional deficiencies within the Mexican population, and public health policies during the pandemic waves.

To avoid reporting bias, studies tracking unrelated clinical conditions on temporomandibular disorders or general zoonotic tracing without nutritional framework contribution were formally excluded from the final evaluation matrix. A summary of the eligible studies and their distribution across these four thematic categories is presented in Table 2.

## 4. Conclusions

Vitamin D deficiency is highly prevalent in the Mexican population and represents an important public health concern because of its potential implications for immune function and overall health. In the context of COVID-19, observational studies have consistently reported an association between low vitamin D status and poorer clinical outcomes. However, evidence from randomized controlled trials remains inconsistent and does not allow definitive conclusions to be drawn regarding the therapeutic efficacy of vitamin D supplementation. Although maintaining adequate vitamin D status is advisable as part of general health promotion, its role as a therapeutic intervention for COVID-19 requires further investigation. Given the high prevalence of vitamin D deficiency and the lack of clinical studies conducted in Mexico, well-designed prospective studies and randomized clinical trials are needed to clarify the impact of vitamin D on COVID-19 outcomes and to generate evidence that can inform future clinical practice and public health strategies in the Mexican population.

## Figures and Tables

**Table 1:** Longitudinal demographic and clinical stratification of confirmed SARS-CoV-2 cases in Mexico from 2020 to 2024 [36].

Variable	2020	2021	2022	2023	2024
Total cases (%)	100	100	100	100	100
Female (%)	49.2	51.5	53.8	54	54.5
Male (%)	50.8	48.5	46.2	46	45.5
Mild (outpatient) (%)	78.5	84.1	92.4	91	94.2
Moderate (hospitalized, non-intubated) (%)	15.3	11.8	5.9	7.2	4.8
Severe/Critical (ICU/intubated) (%)	6.2	4.1	1.7	1.8	1
Mortality (%)	12.8	8.9	2.1	1.5	0.9
Case fatality trend (CFR, %)	12.8	8.9	2.1	1.5	0.9
Hospitalization rate (%)	21.5	15.9	7.6	9	6.8
ICU proportion among hospitalized (%)	28.8	25.9	22.4	20	17.2
Estimated vaccination period coverage	None	Partial rollout	High	High	Very high
Dominant variant period	Wild-type/early lineages	Delta wave	Omicron transition	Omicron subvariants	Endemic circulation

**Table 2:** Methodological Matrix and Thematic Categorization of Reviewed Literature.

Thematic Axis	Study Design/Methodology	Core Focus & Variables Addressed	Key Authors & References
1. Immunomodulation & Pathophysiology	Narrative reviews, basic science, and expert perspectives.	RAS/ACE2 axis, cytokine storm, LL37 peptide, VDR dysfunction, and lung protection.	[2,3,7,9,10,12,18,23,24,28,30,45,46,47,48]
2. Clinical Evidence & Prognosis	Observational studies (cohorts, case–controls) and meta-analyses.	Serum 25(OH)D levels, relative risk, odds ratios (OR), ICU admission, and mortality prediction.	[8,13,14,20,21,26,29,37,42,43,45,49,50,51,52,53,54]
3. Therapeutic Interventions	Randomized controlled trials (RCTs) and therapeutic meta-analyses.	Dosing regimens (UI/day), recovery time, clinical trials, and vaccine immunogenicity.	[5,6,11,16,31,41,43,45,46,51]
4. Public Health & Mexican Perspective	Population surveys, official government databases, and epidemiological reports.	Mexican Official Frameworks, ENSANUT data, lockdown effects, latitude, and socio-demographic disparities.	[4,17,19,32,33,34,35,36,39,41,48,52]

## Data Availability

No new data were created or analyzed in this study. Data sharing is not applicable to this article.

## Abbreviations

The following abbreviations are used in this manuscript:

SARS-CoV-2	Severe Acute Respiratory Syndrome Coronavirus 2
RNA	Ribonucleic Acid
UV	Ultraviolet Radiation
DBP	Vitamin D Binding Protein
VDR	Vitamin D Receptor
NK	Natural Killer Cells
IL	Interleukin
TNF	Tumor Necrosis Factor
IFN	Interferon
TGF	Transforming Growth Factor
RXR	Retinoid X Receptor
VDRE	Vitamin D Response Element
DNA	Deoxyribonucleic Acid
HIV	Human Immunodeficiency Virus
SARS-CoV	Severe Acute Respiratory Syndrome Coronavirus
MERS-CoV	Middle East Respiratory Syndrome Coronavirus
ENSANUT	National Health and Nutrition Survey
IMSS	Mexican Social Security Institute
RAAS	Renin–Angiotensin–Aldosterone System
CUCEI	University Center of Exact Sciences and Engineering
WHO	World Health Organization
